# Occurrence of advance care planning for persons with dementia, cancer and other chronic-progressive diseases in general practice: longitudinal analysis of data from health records linked with administrative data

**DOI:** 10.1136/bmjopen-2024-097655

**Published:** 2025-09-30

**Authors:** Danny Hommel, Bahar Azizi, Mandy Visser, Sascha R Bolt, Jeanet W. Blom, Daisy J A Janssen, Hein P.J. van Hout, Anneke L. Francke, Robert A Verheij, Karlijn J Joling, Jenny T. van der Steen

**Affiliations:** 1 Department of Primary and Community Care and Radboudumc Alzheimer Center, Radboud University Medical Center, Nijmegen, The Netherlands; 2 Groenhuysen Organisation, Roosendaal, The Netherlands; 3 Department of Public Health and Primary Care, Leiden University Medical Center, Leiden, The Netherlands; 4 Topaz, Leiden, The Netherlands; 5 Department of Tranzo, Tilburg School of Social and Behavioral Sciences, Tilburg University, Tilburg, The Netherlands; 6 Department of Health Services Research and Department of Family Medicine, Care and Public Health Research Institute, Faculty of Health Medicine and Life Sciences, Maastricht University, Maastricht, The Netherlands; 7 Department of Research and Development, Ciro, Horn, The Netherlands; 8 Department of General Practice, Amsterdam UMC, Vrije Universiteit Amsterdam, Amsterdam, The Netherlands; 9 Aging & Later Life, Amsterdam Public Health Research Institute, Amsterdam, The Netherlands; 10 NIVEL, Netherlands Institute for Health Services Research, Utrecht, The Netherlands; 11 Department of Public and Occupational Health, Amsterdam UMC, Vrije Universiteit Amsterdam, Amsterdam, The Netherlands; 12 Expertise Center Palliative Care, Amsterdam UMC, Amsterdam, The Netherlands; 13 Department of Medicine for Older People, Amsterdam UMC, Vrije Universiteit Amsterdam, Amsterdam, The Netherlands; 14 Cicely Saunders Institute, King’s College London, London, UK

**Keywords:** Dementia, Primary Care, Electronic Health Records, PALLIATIVE CARE

## Abstract

**Abstract:**

**Objectives:**

There are substantial barriers to initiate advance care planning (ACP) for persons with chronic-progressive disease in primary care settings. Some challenges may be disease-specific, such as communicating in case of cognitive impairment. This study assessed and compared the initiation of ACP in primary care with persons with dementia, Parkinson’s disease, cancer, organ failure and stroke.

**Design:**

Longitudinal study linking data from a database of Dutch general practices’ electronic health records with national administrative databases managed by Statistics Netherlands.

**Setting and participants:**

Data from general practice records of 199 034 community-dwelling persons with chronic-progressive disease diagnosed between 2008 and 2016.

**Outcome measure:**

Incidence rate ratio (IRR) of recorded ACP planning conversations per 1000 person-years in persons with a diagnosis of dementia, Parkinson’s disease, organ failure, cancer or stroke, compared with persons without the particular diagnosis. Poisson regression and competing risk analysis were performed, adjusted for age, gender, migration background, living situation, frailty index and income, also for disease subsamples.

**Results:**

In adjusted analyses, the rate of first ACP conversation for persons with organ failure was the lowest (IRR 0.70 (95% CI 0.68 to 0.73)). Persons with cancer had the highest rate (IRR 1.75 (95% CI 1.68 to 1.83)). Within the subsample of persons with organ failure, the subsample of persons with dementia and the subsample of stroke, a comorbid diagnosis of cancer increased the probability of ACP. Further, for those with organ failure or cancer, comorbid dementia decreased the probability of ACP.

**Conclusions:**

Considering the complexity of initiating ACP for persons with organ failure or dementia, general practitioners should prioritise offering it to them and their family caregivers. Policy initiatives should stimulate the implementation of ACP for people with chronic-progressive disease.

**STRENGTHS AND LIMITATIONS OF THIS STUDY**

- Use of a large sample, including commonly under-represented subgroups such as persons with a migration background.
- Use of routine care data limiting the risk of selection bias.
- Long follow-up period.
- Not all advance care planning conversations and all diagnoses might have been recorded in the electronic health records.
- Due to the nature of the longitudinal data and the need to combine different data sets, older data were used.

## Background

 Advance care planning (ACP) is the process of having conversations about preferences for future healthcare, and may include documentation of these preferences.^1^ ACP enables patients and their family caregivers to discuss values and priorities in future care and medical treatments with healthcare professionals.^1^ ACP can help provide preferred care and treatment and has the potential to increase quality of life and comfort, to increase utilisation of palliative care services, and to reduce hospitalisation.^2^,^5^

Although originating from the context of palliative care for persons with cancer, ACP is currently recommended in guidelines for chronic-progressive diseases, including dementia,^6^ Parkinson’s disease,^7 8^ organ failure^9 10^ and stroke.^11^ Despite being included in guidelines, ACP is often not optimally applied in practice.^12^,^14^ In a previous study among persons with dementia, we found only 22 first ACP conversations per 1000 person-years of follow-up in health records in general practice in the Netherlands.^15^ The frequency of ACP within other disease groups is reported in the literature, and although studies mostly focus on a single or a few diseases with a limited sample, they indicate that there are disparities in ACP conversations between disease groups.^16^,^18^ In patients with cancer, the reported occurrence of ACP discussions with healthcare providers ranged from 4% in an Australian study to 62% in a US study.^19^,^23^ Among patients with organ failure, documented advance directives ranged from 4% to 51%.^24^,^28^ A Canadian study suggested that ACP conversations barely occurred in stroke patients.^17^ The occurrence of ACP discussions with patients with Parkinson’s disease ranged from 10% in a UK study to 47% in a Dutch study.^29 30^ These studies presented highly varied estimates and did not compare disease groups. Recently, a small Dutch study compared disease groups and suggested that the prevalence of ACP in persons with cancer is higher (84%), compared with persons with organ failure (57%) and persons with multimorbidity (42%).^16^

In the current study, we examine the rates of first ACP conversation in multiple chronic-progressive diseases that are prevalent causes of disability in the general population.^31^ Among these diseases are three chronic-progressive diseases: dementia, Parkinson’s disease and organ failure (comprising heart failure, kidney disease and chronic obstructive pulmonary disease), and two diseases that can result in chronic disability: cancer and stroke. In line with previous studies, we expected ACP conversations to be most common in persons with cancer. We expected the least documented ACP conversations to be with patients with dementia, because dementia typically involves cognitive decline and substantial prognostic uncertainty.^13 14^ By studying differences between disease groups, we can identify possible inequalities in access to ACP conversations. Underserved populations of people with particular diseases, such as diseases that involve communication problems, may be targeted for interventions to promote ACP in general practice.

## Methods

### Study aim, design and setting

To assess and compare the initiation of ACP in primary care with persons with dementia, Parkinson’s disease, cancer, organ failure and stroke, we used longitudinal electronic health record (EHR) data from general practices in the Netherlands. The general practitioner (GP) in the Netherlands acts as the gatekeeper to specialist care and is typically involved in chronic diseases for community-dwelling patients.^32^ Patients residing in nursing homes or other long-term care facilities are typically not under the care of GPs, but receive medical care from so-called ‘elderly care physicians’, specialised in care for people who are older, frail and/or have complex chronic care needs.^33^ Therefore, nursing home residents were not included in this study. The occurrence and date of the ACP conversation were registered as an ICPC code in the EHR system of the general practice. Physicians documented these conversations using the ICPC codes A20 (labelled ‘request/conversation about euthanasia’) and A58 (labelled ‘ACP’). While the A20 ACP code had originally been used for conversations on euthanasia, the Dutch College of General Practitioners advised using it to record any ACP conversation.^34 35^

### Data sources

The EHR data are part of the NIVEL Primary Care Database (PCD).^36 37^ The NIVEL-PCD embodies health records of—at the time of this study—451 general practices, which means it is representative of Dutch general practices in terms of patients’ age and sex, practice size, and geographical distribution of patients. NIVEL-PCD offers support to GPs with the coding of consultations, and GPs are reimbursed for participation based on the quality of their recording.^38 39^ The EHR data were complemented with demographic data from the mandatory nationwide administrative databases managed by Statistics Netherlands (Centraal Bureau voor de Statistiek, CBS), that is, the population registers of the municipalities and tax authorities. These include sociodemographic characteristics, date of death, household data and income registration.

### Data linkage

The relevant data from the data sources were linked to create the data set for analysis. After pseudonymisation of EHR data, the data were transferred to the remote access secured environment of CBS for data linkage. Pseudonyms were created by a Trusted Third Party based on the citizen service number or a combination of birth date, sex and zip code. In total, 91.1% of the data was successfully matched. A small sample of 2.0% had multiple health records, possibly due to changing GPs during the course of the disease.

### Case selection

**Figure 1:**
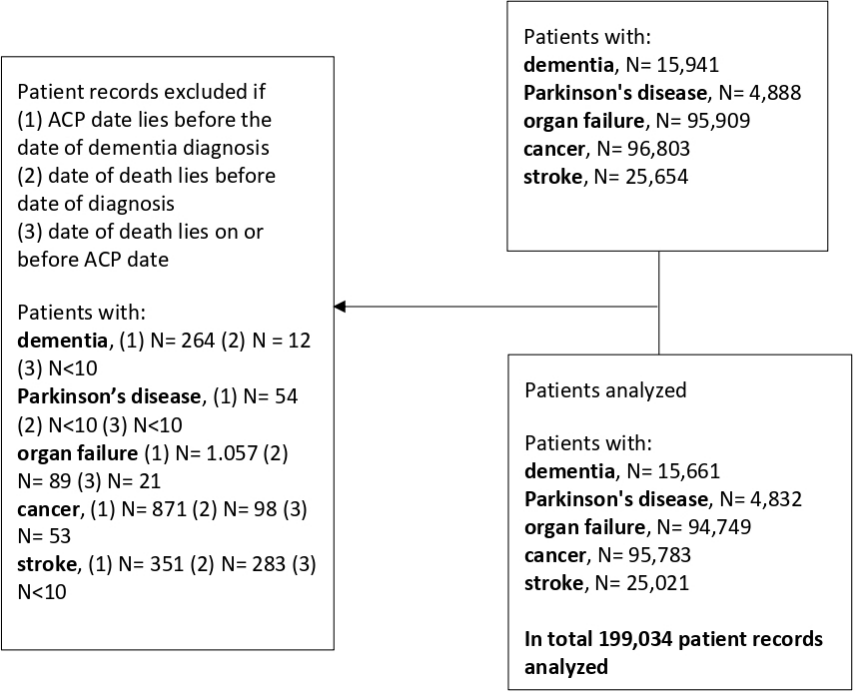
Flow chart of the selection of the study sample. ACP, advance care planning.

For the current analysis, we selected data of persons with at least one of the chronic-progressive diseases of dementia, Parkinson’s disease, organ failure (heart failure, kidney disease and chronic obstructive pulmonary disease), cancer and stroke. Diagnoses were determined by disease-specific registrations of the GP or a medical specialist, which were recorded in the EHR system of the general practice using codes following the International Classification of Primary Care (ICPC-1).^40^ ICPC (sub)codes were selected for each disease (online supplemental additional file 1 table 1). ICPC subcodes are not registered in the NIVEL-PCD, and we could therefore not select kidney failure (U99.01). We used the primary ICPC code U99, which includes kidney failure. In addition to the diagnosis code, physicians documented the date of diagnosis. We included data of persons who were born before or in 1965 with a recorded diagnosis in the years 2008–2016. Persons with a diagnosis before 2008 or after 2016 were excluded, as no information on ACP conversations was available for these years. We excluded persons for whom the date of their first ACP conversation was recorded before the date of the particular diagnosis. This served to compare disease groups regarding ACP that potentially considered the particular diagnosis and, with no diagnosis yet, we could not select the case for the purpose of our study. Also, we excluded registration errors, such as when the recorded date of death was before the date of diagnosis or before the date of the first recorded ACP conversation (figure 1).

### Outcome measure

The outcome measure for the analysis was the incidence rate ratio (IRR) of recorded ACP conversations per 1000 person-years in persons with a diagnosis (dementia, Parkinson’s disease, organ failure, cancer and stroke), compared with persons with any of the other diagnoses. As a competing outcome, we examined mortality.

### Covariates

Covariates were age, sex, migrant status, income, living situation and frailty score (online additional file 1, table 2 shows data sources). The date of the diagnosis was the reference date for these variables. The variable ‘migrant status’ consisted of the following categories: non-Western migration background (combining Surinamese, Antillean, Aruban, Moroccan, Turkish and other non-Western migration backgrounds) and Western background (a native Dutch background or Western migration background). Age categories were: age under 65 years, 65–74, 75–84 and 85 years and above. As an income measure, we chose the income of the primary breadwinner of the household, as this resulted in the least missing values. The living situation was categorised as living with one or more cohabitants, living alone, or living in an institution.

In addition, we derived frailty scores from the EHR data. To calculate the frailty index, we screened EHR data for 35 predefined clinically relevant health problems, defined as ‘health deficits’. Every health deficit represents a number of ICPC codes.^41 42^ Each ICPC code represents a symptom or disease. If one or more of the ICPC codes representing a health deficit were recorded in the patient’s EHR, they received a score of 1 for that health deficit. All scores of the 35 health deficits were then summed for each patient and divided by the total number of possible health deficits (35) to determine individual frailty index scores. Consistent with previous studies, we classified the frailty index score as: non-frail (three or fewer health deficits: frailty index score ≤0.08), prefrail (four to eight health deficits: 0.08 <frailty index score <0.25) and frail (nine or more health deficits: frailty index score ≥0.25).^43^,^46^

### Statistical analysis

We used descriptive statistics to present the characteristics of patient groups per disease (dementia, Parkinson’s disease, organ failure, cancer and stroke). Missing data analysis showed that the percentage of missing data was <1% for all variables, except for income, for which the percentage of missing income ranged from 1% to 4%. The rate of having a first recorded ACP conversation was calculated per 1000 person-years. In order to compare the impact of diseases on the incidence rate of ACP, IRRs were calculated using Poisson regression. Poisson regression is a regression analysis for count and rate data. It allows for adding denominators in the Poisson regression modelling in the form of offsets. The denominator could also be the unit time of exposure, such as person-years,^47^ and was therefore appropriate for the analysis in this study. In the Poisson model, ratios were adjusted for age, gender, migration background, living situation and income. However, this analysis did not allow for an interpretation of the disease-specific effect within the course of a specific disease trajectory (eg, to examine whether cancer is associated with an increased ACP rate in persons with dementia). Also, Poisson regression ignores the impact of mortality. Because death alters the probability of engaging in ACP (persons who die before ACP can no longer engage in ACP), it was considered a competing risk. Therefore, we additionally performed competing risk analyses per disease group with all other chronic diseases and covariates included in the model.^48^ The significance level for these analyses was set at 0.05. SPSS V.25 was used for descriptive analyses. Competing risk analyses were performed using R Studio (R V.4.3.0), with the use of the package ‘cmprsk’.

### Patient and public involvement

Patient representatives were involved in an advisory committee. They advised us about the conduct of the study and supported us in interpreting and disseminating the study findings.

## Results

### Study sample

In total, 199 034 persons with at least one of the chronic diseases of interest were identified and included for analysis (figure 1). The largest disease group involved cancer (n=95 783), followed by persons with organ failure (n=94 749). The samples of other diagnoses also numbered in the thousands, with 25 021 persons with stroke, 15 661 persons with dementia and 4832 persons with Parkinson’s disease. The majority of persons with dementia were female (n=9903; 63%), while most persons with Parkinson’s disease were male (n=2799; 58%) (table 1). For other groups, the number of males versus females was roughly equal. Persons with cancer were the youngest on average (mean age at diagnosis 67.9; SD 11.1), followed by persons with organ failure (mean age 71.3; SD 11.8) and persons with stroke (mean age 71.3; SD 11.8). Persons with Parkinson’s disease (mean age 73.2; SD 9.5) and persons with dementia (mean age 80.5; SD 8.2) were older. For all disease groups, most persons lived at home with one or more cohabitants (ranging from 56% to 72% between disease groups). Also, for all disease groups, the majority of persons were native Dutch (ranging from 86% to 88% between disease groups). Most persons had a frailty score in the ‘prefrail’ range (ranging from 54% to 73% between disease groups).

**Table 1:** Characteristics of the patients (n=199 034)

	Patients with dementia (n=15 661)	Patients with Parkinson’s disease (n=4832)	Patients with organ failure (n=94 749)	Patients with cancer (n=95 783)	Patients with stroke (n=25 021)
	n (%)	n (%)	n (%)	n (%)	n (%)
Female	9903 (63)	2033 (42)	49 291 (52)	49 883 (52)	12 362 (49)
Age at diagnosis, mean (SD)	80.5 (8.2)	73.2 (9.5)	71.3 (11.8)	67.9 (11.1)	71.3 (11.8)
Under 65 years	743 (5)	937 (19)	29 918 (32)	38 840 (41)	7774 (31)
65–74 years	2698 (17)	1629 (34)	25 785 (27)	29 999 (31)	6874 (28)
75–84 years	7335 (47)	1820 (38)	26 749 (28)	20 629 (22)	7196 (29)
85 years and above	4885 (31)	446 (9)	12 298 (13)	6315 (7)	3177 (13)
Living situation					
With one or more cohabitants	8753 (56)	3438 (71)	60 261 (64)	69 132 (72)	16 129 (64)
Alone	6908 (44)	1394 (29)	34 488 (36)	26 651 (28)	8892 (36)
In an institution	1683 (11)	266 (6)	3633 (4)	1602 (2)	1090 (4)
Migrant status					
Native Dutch	13 668 (87)	4234 (88)	81 123 (86)	84 839 (89)	21 471 (86)
Western migration background	1545 (10)	431 (9)	9394 (10)	8523 (9)	2423 (10)
Surinamese/Antillean/Aruban	189 (1)	50 (1)	1588 (2)	856 (1)	448 (2)
Moroccan/Turkish	178 (1)	85 (2)	1591 (2)	856 (1)	372 (2)
Other non-Western	80 (1)	32 (1)	1041 (1)	697 (1)	668 (3)
Frailty index (0–1), median (range)	0.11 (0.46)	0.11 (0.34)	0.11 (0.46)	0.09 (0.43)	0.11 (0.46)
Mean (SD)	0.12 (0.06)	0.11 (0.06)	0.11 (0.06)	0.09 (0.05)	0.11 (0.06)
Non-frail (%)	3752 (24)	1433 (30)	24 089 (25)	42 678 (45)	6665 (27)
Prefrail	11 401 (73)	3257 (67)	68 076 (72)	51 673 (54)	17 688 (71)
Frail	508 (3)	142 (3)	2584 (3)	1249 (1)	668 (3)
Household income in**,** mean (SD)	25 739 (21 249)	27 628 (22 823)	25 544 (24 475)	29 301 (36 210)	26 099 (17 704)
Median (range)	21 240 (720 559)	23 324 (638 513)	21 961 (4 259 669)	24 983 (6 375 139)	22 604 (1 417 072)

### Rate of first ACP conversation

Between 2008 and 2016, the first ACP conversation was initiated with 9485 persons (4.8%). Per disease group, the incidence rate of persons with a first ACP conversation ranged from 13.8 per 1000 person-years for persons with organ failure to 19.0 per 1.000 person-years for persons with dementia or Parkinson’s disease. Adjusted for covariates, persons with cancer had a higher rate of ACP (IRR 1.75 (95% CI 1.68 to 1.83)) compared with persons with any of the other diagnoses. Persons with dementia (IRR 0.78 (95% CI 0.73 to 0.84)), organ failure (IRR 0.70 (95% CI 0.68 to 0.73)) or stroke (IRR 0.87 (95% CI 0.82 to 0.93)) had a lower rate of ACP compared with persons with any of the other diagnoses. Persons with Parkinson’s disease had a comparable rate of ACP as persons with other diagnoses (IRR 0.99 (95% CI 0.88 to 1.12)) (table 2).

**Table 2:** First advance care planning conversations recorded in the period 2008–2016 per disease

	n (%)	Unadjusted incidence rate*	Adjusted incidence rate ratio (95% CI)†
Persons with dementia	817 (5.2)	19.0	0.78 (0.73 to 0.84)
Persons with Parkinson’s disease	294 (6.1)	19.0	0.99 (0.88 to 1.12)
Persons with organ failure	4122 (4.4)	13.8	0.70 (0.68 to 0.73)
Persons with cancer	5388 (5.6)	17.4	1.75 (1.68 to 1.83)
Persons with stroke	1120 (4.8)	14.3	0.87 (0.82 to 0.93)

* Incidence rate per 1000 person-years.

† Poisson regression model adjusted for age, gender, migration background, living situation and income differences.

### Comorbid conditions within disease groups

Within the subsample disease groups, organ failure was the most frequent comorbid condition (table 3). The percentage of persons with organ failure varied between 19% in the subsample cancer and 29% in the subsample dementia. The subsample dementia also had the highest percentage of comorbid Parkinson’s disease (3% versus Parkinson’s as a comorbid condition with stroke 2%, organ failure 1% and cancer 1%) and stroke (12% versus Parkinson’s disease as a comorbid condition with stroke 9%, organ failure 7% and cancer 5%), while they had the lowest percentage of comorbid cancer (16% versus cancer as a comorbid condition with stroke 18%, Parkinson’s disease 19% and organ failure 20%).

**Table 3:** Comorbid conditions within disease groups

	Patients with dementia (n=15 661)	Patients with Parkinson’s disease (n=4832)	Patients with cancer (n=95 783)	Patients with organ failure (n=94 739)	Patients with stroke (n=25 021)
	N (%)	N (%)	N (%)	N (%)	N (%)
Comorbid dementia	–	503 (10)	2463 (3)	4593 (5)	1839 (7)
Comorbid Parkinson’s disease	499 (3)	–	900 (1)	1056 (1)	440 (2)
Comorbid cancer	2444 (16)	901 (19)	–	18 439 (20)	4513 (18)
Comorbid organ failure	4883 (29)	1075 (22)	18 499 (19)	–	6691 (27)
Comorbid stroke	1863 (12)	450 (9)	4582 (5)	6750 (7)	–

### Impact of comorbid chronic disease during disease courses of other diseases on ACP conversations

Cancer was associated with a shorter time to ACP conversations in three subsamples (table 4). In the subsample of dementia, the HR of a comorbid diagnosis of cancer was 1.37 (95% CI 1.15 to 1.62). Comorbid cancer also increased the chance of ACP initiation in the subsample organ failure (HR 1.76 (95% CI 1.65 to 1.89)) and the subsample stroke (HR 1.64 (95% CI 1.44 to 1.88)).

**Table 4:** Impact of diseases during the course of another disease on the time of the first ACP conversation*

Persons with dementia (n=15 127; 534 cases omitted due to missing values in covariates)		
	HR†	95% CI
Parkinson’s disease	1.03	0.71; 1.51
Organ failure	1.07	0.91; 1.26
Cancer	1.37‡	1.15; 1.62
Stroke	1.08	0.89; 1.33
Persons with Parkinson’s disease (n=4762; 70 cases omitted due to missing values in covariates)		
	HR	95% CI
Dementia	0.82	0.56; 1.21
Organ failure	0.83	0.63; 1.11
Cancer	1.12	0.88; 1.57
Stroke	0.91	0.62; 1.36
Persons with organ failure (n=93 591; 1158 cases omitted due to missing values in covariates)		
	HR	95% CI
Dementia	0.86‡	0.76; 0.97
Parkinson’s disease	0.85	0.65; 1.12
Cancer	1.76‡	1.65; 1.89
Stroke	0.97	0.88; 1.08
Persons with cancer (n=94 919; 864 cases omitted due to missing values in covariates)		
	HR	95% CI
Dementia	0.83‡	0.72; 0.96
Parkinson’s disease	0.94	0.73; 1.20
Organ failure	1.10‡	1.02; 1.18
Stroke	1.10	0.98; 1.22
Persons with stroke (n=24 689; 332 cases omitted due to missing values in covariates)		
	HR	95% CI
Dementia	0.82	0.66; 1.01
Parkinson’s disease	0.99	0.66; 1.47
Organ failure	0.95	0.83; 1.09
Cancer	1.64‡	1.44; 1.88

* Results of competing risk analysis adjusted for difference in age, gender, migration background, living situation, frailty index and income.

† HR >1 indicates shorter time to first ACP. HR <1 indicates longer time to first ACP.

‡ P value <0.05.

ACP, advance care planning.

In contrast, people with comorbid dementia had a shorter time to ACP conversation compared with dementia alone in two subsamples; in the subsamples, organ failure (HR 0.86 (95% CI 0.76 to 0.97)) and cancer (HR 0.83 (95% CI 0.72 to 0.96)). Within the subsample of persons with cancer, organ failure increased the time to ACP initiation (HR 1.10 (95% CI 1.02 to 1.18)).

## Discussion

This large study using EHRs of GPs linked with national administrative databases compares—for the first time—the rates of first ACP conversations of five disease groups. In adjusted analyses, the rate for persons with organ failure was the lowest, followed by persons with dementia. Persons with cancer had the highest rate of ACP conversations. Within the subsample of persons with organ failure, a comorbid diagnosis of cancer increased the probability of ACP. This pattern was similar for the subsamples of dementia and stroke. Further, in the subsamples of organ failure and cancer, comorbid dementia decreased the probability of ACP.

Adjusted for covariates, persons with cancer had the highest IRR, reflecting a rate of ACP nearly twice that of other diseases. This confirms the findings of a recent small study that reported the prevalence of ACP in persons with cancer at 84%, compared with 57% for persons with organ failure and 42% for persons with multimorbidity.^16^ Such large differences are difficult to explain as many known barriers to ACP are not disease-specific (eg, lack of time, lack of training and fear of diminishing patients’ hope).^49^ Specific triggers to initiate ACP conversations can be disease-specific, for example, for persons with cancer, ACP is often initiated when no curative treatments are available. In addition, GPs initiate ACP conversations closer to death in persons with organ failure or multimorbidity, compared with persons with cancer.^50^ For persons with cancer, triggers for ACP are associated with the ‘timeline of disease’ (eg, diagnosis, no curative treatments available or start of treatments and diagnostics). For persons with organ failure and multimorbidity, triggers of ACP are mostly associated with ‘symptoms indicating deterioration’. When based on symptoms of deterioration, GPs’ awareness of the need for ACP typically arises gradually and relatively late, with the risk of being too late.^18 49^ For example, when the initiation of ACP is postponed to admission to a nursing home, severe cognitive impairment complicates the involvement of the person himself or herself, which has been identified as good practice in ACP for people with dementia.^51^,^53^ Postponed to nursing home admission, the person with dementia is deprived of the opportunity to make decisions for himself or herself. In general practice, however, there is no such natural moment to initiate ACP, such as the routine ACP conducted on nursing home admission. GPs may also wait until a critical stage because they fear that earlier ACP might decrease the patient’s hope for the future and negatively affect the doctor–patient relationship.^54^ However, a majority of persons with chronic diseases prefer an earlier ACP conversation.^55^

In the adjusted analysis, dementia decreased the chance of ACP initiation in multiple disease groups. The literature reports numerous barriers to ACP in dementia.^5 56^ First, the timing of ACP is perhaps even more challenging, as the window of opportunity for initiating ACP for persons with dementia is smaller than in other diseases.^5^ Second, communicating with persons with dementia might require additional communication skills that perhaps not all GPs have mastered.^51 56^ For example, persons with dementia feel more uncertain in making treatment decisions due to decreasing cognitive capacity. Additional communication strategies to bolster the decision-making capacity are needed but require additional skill and time. Also, GPs may unjustly fear overestimating the decision-making capacity of persons with dementia, as they are generally not trained in in-depth clinical or neuropsychological assessments.^57^

### Strengths and limitations

This study is the first direct comparison of ACP initiation in five major disease groups, using the largest data set in the Netherlands with data on ACP. These data also include data from commonly under-represented subgroups, such as persons with a migration background. Other strengths of this study are the long and complete follow-up with low numbers of missing data. Several limitations should also be mentioned. Registry and administrative databases suffer from an inherent problem that the measurements were not specifically designed for research purposes. As a result, perhaps not all ACP conversations and all diagnoses were recorded in the database. Diagnoses prior to 2008 (and no recent follow-up) were missing. As a result, persons with less severe disease that did not warrant a recent GP consultation could be under-represented. Further, due to the nature of the longitudinal data and the need to combine different data sets, older data (up to 2016) had to be used. However, the findings are in line with smaller studies that used more recent health records in Dutch primary care.^16^

## Conclusions

Dutch GPs initiate ACP less frequently for persons with dementia, stroke and organ failure, compared with persons with cancer. Considering the complexity of initiating ACP in persons with organ failure or dementia, GPs may prioritise offering it to them and their family caregivers. Practice improvement initiatives should stimulate implementation of ACP with chronic-progressive disease, for example, by reimbursing time to conduct ACP conversations. Also, guidelines addressing the treatment of chronic-progressive diseases can pay more attention to ACP. Already available tools to support healthcare professionals in addressing palliative care needs can be helpful as well.^58 59^

## Supplementary material

10.1136/bmjopen-2024-097655 online supplemental file 1

## Data Availability

Data may be accessed at CBS and are not publicly available.
