# Supplementary material for: Occurrence of advance care planning for persons with dementia, cancer and other chronic-progressive diseases in general practice: longitudinal analysis of data from health records linked with administrative data
Source: BMJ Open. 2025 Sep 30;15(9):e097655. doi: 10.1136/bmjopen-2024-097655 (PMC12496107; doi:10.1136/bmjopen-2024-097655)
Supplement: online supplemental file 1 [file bmjopen-15-9-s001.docx]

**Additional file 1**

**Table 1. Diseases and ICPC codes**

| **Disease** | **ICPC** |
| --- | --- |
| **Dementia** | P70 |
| **Parkinson’s disease** | N87 |
| **Cancer (various types)** |  |
| *Other malignancy reproductive system /breast male* | Y78 |
| *Malignancy breast female* | X76 |
| *Malignancy nervous system* | N74 |
| *Malignancy prostate* | Y77 |
| *Malignancy bladder* | U76 |
| *Malignancy thyroid* | T71 |
| *Malignancy pancreas* | D76 |
| *Malignancy stomach* | D74 |
| *Malignancy related to pregnancy* | W72 |
| *Other malignancy urinary system* | U77 |
| *Malignancy kidney* | U75 |
| *Malignancy colon/rectum* | D75 |
| *Malignancy with unknown primary location* | A79 |
| *Malignancy cervix uteri* | X75 |
| *Malignancy bronchus/pulmonary* | R84 |
| *Other malignancy blood/lymphatic system* | B74 |
| *Malignancy skin/subcutis* | S77 |
| *Other malignancy airways* | R85 |
| *Other malignancy reproductive system female* | X77 |
| *Other/not otherwise specified malignancy digestive organs* | D77 |
| *Leukemia* | B73 |
| *Neoplasm eyes/adnexen* | F74 |
| *Neoplasm ears* | H75 |
| *Neoplasm heart and vascular system* | K72 |
| *Neoplasm musculoskeletal system* | L71 |
| *Malignancy bronchus/pulmonary* | R84 |
| **Organ failure** |  |
| *Congestive heart failure* | K77 |
| *Other urinary tract disease(s)* | U99 |
| *COPD* | R95 |
| **Stroke** | K90 |

**Table 2. Data sources of outcome measures and covariates**

| **Variables** | **Source** | **Period** |
| --- | --- | --- |
| Outcome measure |  |  |
| Advance care planning (ICPC codes A20 and A58) | NIVEL-PCD | 2008-2016 |
| Covariates |  |  |
| Frailty index* | NIVEL-PCD | at or year prior to date of diagnosis |
| Age | CBS; BasisRegistratie Personen | at time of diagnosis |
| Gender | CBS; BasisRegistratie Personen | at time of diagnosis |
| Migrant status | CBS; BasisRegistratie Personen | at time of diagnosis |
| Living situation | CBS; Huishoudensregister | at time of diagnosis |
| Income | CBS; Integraal huishoudens inkomen | at time of diagnosis |
| Death date | CBS; Overlijdensregister | 2008-2016 |

*Calculated using algorithm published in Drubbel I, et al. J Gerontol A Biol Sci Med Sci 2013;68(3):301-8
