# Supplementary material for: Occurrence of advance care planning for persons with dementia, cancer and other chronic-progressive diseases in general practice: longitudinal analysis of data from health records linked with administrative data
Source: BMJ Open. 2025 Sep 30;15(9):e097655. doi: 10.1136/bmjopen-2024-097655 (PMC12496107; doi:10.1136/bmjopen-2024-097655)
Supplement: Supplementary file 2 [file bmjopen-2024-097655.reviewer_comments.pdf]

PEER REVIEW HISTORY

BMJ Open publishes all reviews undertaken for accepted manuscripts. Reviewers are asked to complete a checklist review form and are provided with free text boxes to elaborate on their assessment. These free text comments are reproduced below.

ARTICLE DETAILS

Title (Provisional)

Occurrence of advance care planning for persons with dementia, cancer and other chronic-progressive diseases in general practice: Longitudinal analysis of data from health records linked with administrative data

Authors

Hommel, Danny; Azizi, Bahar; Visser, Mandy; Bolt, Sascha R.; Blom, Jeanet; Janssen, Daisy J.A.; van Hout, Hein; Francke, Anneke; Verheij, Robert A; Joling, Karlijn J.; van der Steen, Jenny Theodora

---

VERSION 1 - REVIEW

**Reviewer**	**1**
**Name**	**Stevens, Julie**
**Affiliation**	**Vrije Universiteit Brussel**
**Date**	**14-Feb-2025**
**COI**	**None**

This is a comprehensive study of general practice records from Dutch general practices, which is strengthened by its use of a representative database.

Overall I think the manuscript is written well and concisely and the results are clearly presented.

The analyses regarding advance care planning in different comorbidities is an especially important insight, which brings to the forefront a reality of clinical practice that sometimes goes underrepresented in disease-specific ACP research: many patients have comorbid conditions or comorbid frailty, contributing to additional complexity navigating the initiation of ACP.

Just a few comments below which I hope can be helpful to the authors.

Abstract:

In the outcome measure, phrasing is unclear: “in persons with a diagnosis […] compared to persons with any of the other diagnoses”. What other diagnoses are meant?

Introduction:

Introduction:

While the introduction provides a good overview of studied rates of ACP for different illness populations in different countries, the research question could be developed more strongly. There is an argument that limited disease populations studied or small samples are limitations of previous work, so there is some implicit referral to the need for the current research. I would appreciate if the authors could concisely make the case for also comparing disease groups, and what the expected contribution to the literature will be of addressing this question.

Method:

There is mention here that patients in nursing homes and long-term care are usually not under the care of GPs. My immediate thought on reading this was that patients with illnesses related to cognitive decline might be represented in the populations residing in these settings, and thus information about ACP initiation (if any) in nursing homes and long-term care could further have contributed to understanding how ACP is offered to people with conditions such as dementia and Parkinson's disease. For instance, in Belgium, more and more frequently nursing homes discuss ACP at intake as a standard of practice (and sometimes a GP is the person doing this). Is anything like this being done in the Netherlands? This isn't necessarily an issue for the questions being asked in the current manuscript, if the population of interest specifically is patients in primary care, but having this information could broaden our understanding. Possibly an area for additional research?

Can the authors elaborate for the rationale of including data of persons born up to and including the year 1965?

Some additional description of statistical methods for outcomes related to time to first ACP (as opposed to incidence of first ACP) might be useful here.

In the case selection, there is mention of excluding cases where the first ACP conversation was recorded prior to diagnosis; in Figure 1, this is specifically dementia diagnosis?

Results:

For comprehensiveness, could the authors include in-text percentages for most persons living at home with cohabitants, being native Dutch, and having a pre-frail range frailty score?

Discussion:

For those unfamiliar with the ICPC codes, can the authors elaborate on any further details for the codes A20 and A58? I see that the authors interpret the findings as GPs initiating ACP more or less often within certain illness populations, but this is a one-directional interpretation. For the code A20 at least, I would presume this is patient-initiated since it involves a request. The dataset may not allow for interpretation of who, patient or GP, is the

one initiating the conversation; if this is the case, I think it should be acknowledged as a limitation, and perhaps the concluding statement could be amended to not place the onus of a lack of initiation fully on GPs? Though of course GPs should still be expected to respond appropriately if a patient initiates.

The conclusion refers to policy initiatives stimulating implementation of ACP in chronic progressive disease. It would strengthen the discussion if the authors could provide an example or two. Maybe there is some evidence in the literature of initiatives that have been successful in other countries? This could be taken into the discussion section with some implications/recommendations for practice and policy, as currently the recommendation is quite general.

VERSION 1 - AUTHOR RESPONSE

Reviewer 1: Dr. Julie Stevens, Vrije Universiteit Brussel

1. This is a comprehensive study of general practice records from Dutch general practices, which is strengthened by its use of a representative database. Overall I think the manuscript is written well and concisely and the results are clearly presented. The analyses regarding advance care planning in different comorbidities is an especially important insight, which brings to the forefront a reality of clinical practice that sometimes goes underrepresented in disease-specific ACP research: many patients have comorbid conditions or comorbid frailty, contributing to additional complexity navigating the initiation of ACP. Just a few comments below which I hope can be helpful to the authors.

Response:
Thank you for appreciating our study.

2. Abstract: In the outcome measure, phrasing is unclear: “in persons with a diagnosis […] compared to persons with any of the other diagnoses”. What other diagnoses are meant?

Response and revision:
We can understand that this sentence can be unclear. We revised the sentence to: *“in persons with a diagnosis of dementia, Parkinson's disease, organ failure, cancer or stroke, compared to persons without the particular diagnosis.” [Abstract, lines 48-49]*

3. Introduction: While the introduction provides a good overview of studied rates of ACP for different illness populations in different countries, the research question could be developed more strongly. There is an argument that limited disease populations studied or small samples are limitations of previous work, so there is some implicit referral to the need for the current research. I would appreciate if the authors could concisely make the case for also comparing disease groups, and what the expected contribution to the literature will be of addressing this question.

Response and revision:
Thank you for your suggestion to provide a stronger rationale for comparing disease groups.
We revised lines 94-96:

[15] The frequency of ACP within other disease groups is reported in the literature, ~~but~~ and although studies mostly focus on a single or a few diseases with a limited sample, they indicate that there are disparities in ACP conversations between disease groups.[16-18]

Further, to lines 112-115, we added: *"By studying differences between disease groups, we can identify possible inequalities in access to ACP conversations. Underserved populations of people with particular diseases such as diseases that involve communication problems may be targeted for interventions to promote ACP in general practice.*

4. Method: There is mention here that patients in nursing homes and long-term care are usually not under the care of GPs. My immediate thought on reading this was that patients with illnesses related to cognitive decline might be represented in the populations residing in these settings, and thus information about ACP initiation (if any) in nursing homes and long-term care could further have contributed to understanding how ACP is offered to people with conditions such as dementia and Parkinson's disease. For instance, in Belgium, more and more frequently nursing homes discuss ACP at intake as a standard of practice (and sometimes a GP is the person doing this). Is anything like this being done in the Netherlands? This isn't necessarily an issue for the questions being asked in the current manuscript, if the population of interest specifically is patients in primary care, but having this information could broaden our understanding. Possibly an area for additional research?

Response:
Thank you for your consideration. Indeed, in the Netherlands, ACP is conducted upon admission to a nursing home as a standard of practice. Certified "elderly care physicians" who are on the staff of nursing homes are responsible for initiating ACP. However, this is often late in the disease trajectory of chronic-progressive disease. Therefore, this study focusses specifically at initiation of ACP in primary care. We have attempted to better explain and underpin this in the Methods and Discussion.

Revisions:
To explain why nursing home residents in the Netherlands usually don't receive care from the GP, the following sentence, "Patients residing in nursing homes or other long-term care facilities are typically not under the care of GPs.", has been revised into: *"Patients residing in Dutch nursing homes or other long-term care facilities are typically not under the care of GPs, but receive medical care from so called elderly care physicians, specialized in care for people who are older, frail and/or have complex chronic care needs.[33] Therefore, nursing home residents were not included this study." (lines 124-126)*

In the Discussion, to this sentence *"GPs awareness of the need for ACP typically arises gradually and relatively late, with the risk of being too late[18,50]"* we added: *"For example, when initiation of ACP is postponed to admission to a nursing home, severe cognitive impairment complicates the involvement of the person him or herself which has been identified as good practice in ACP for people with dementia[51-53]. Postponed to nursing home admission, the person with dementia is deprived of the opportunity to make decisions for him or herself. In general practice, however, there is not such a natural moment to initiate ACP such as the routine ACP conducted upon nursing home admission." [lines 318-323]*

5. Can the authors elaborate for the rationale of including data of persons born up to and including the year 1965?

Response:

The dataset was originally built for a project that aimed to give insight in care trajectories of people with dementia, see e.g. Joling 2020[43]. As people are usually older when being diagnosed with dementia, and due to data minimization arguments, the research team decided to request EHR data from people with birth year 1965 (meaning the youngest persons were at least 43 years in the period of the recorded data). In this way, the study covered the overwhelming majority of people with dementia without requesting too much data.

6. Some additional description of statistical methods for outcomes related to time to first ACP (as opposed to incidence of first ACP) might be useful here.

Response:
As described under "Outcome measure", the outcome measure for the analysis was the incidence rate ratio of recorded ACP conversations per 1,000 person-years in persons with a diagnosis (dementia, Parkinson's disease, organ failure, cancer and stroke), compared to persons with any of the other diagnoses. Poisson regression is a regression analysis for count and rate data. It allows for adding denominators in the Poisson regression modelling in the form of offsets. The denominator could also be the unit time of exposure, such as person-years[47], and was therefore appropriate for the analysis in this study.

Revision:
We have added this information in the statistical analysis section (lines 204-207):
"Poisson regression is a regression analysis for count and rate data. It allows for adding denominators in the Poisson regression modelling in the form of offsets. The denominator could also be the unit time of exposure, such as person-years[47], and was therefore appropriate for the analysis in this study."

7. In the case selection, there is mention of excluding cases where the first ACP conversation was recorded prior to diagnosis; in Figure 1, this is specifically dementia diagnosis?

Response:
We excluded persons for whom the date of their first ACP conversation was recorded before the date of the particular diagnosis, because we aimed at comparing disease groups regarding ACP that potentially considered the particular diagnosis. With no diagnosis yet, we could not select the case for the purpose of our study. This was not unique for the dementia diagnosis; we followed the same procedure for the other disease groups.

Revisions: We revised the following sentence in the Methods (revisions underlined):
We excluded persons for whom the date of their first ACP conversation was recorded before the date of the particular diagnosis. This served to compare disease groups regarding ACP that potentially considered the particular diagnosis and, with no diagnosis yet, we could not select the case for the purpose of our study. (lines 165-167)

8. Results: For comprehensiveness, could the authors include in-text percentages for most persons living at home with cohabitants, being native Dutch, and having a pre-frail range frailty score?

<u>Response</u>:
We have added the percentages in the text (lines 252-255).

9. Discussion: For those unfamiliar with the ICPC codes, can the authors elaborate on any further details for the codes A20 and A58? I see that the authors interpret the findings as GPs initiating ACP more or less often within certain illness populations, but this is a one-directional interpretation. For the code A20 at least, I would presume this is patient-initiated since it involves a request. The dataset may not allow for interpretation of who, patient or GP, is the one initiating the conversation; if this is the case, I think it should be acknowledged as a limitation, and perhaps the concluding statement could be amended to not place the onus of a lack of initiation fully on GPs? Though of course GPs should still be expected to respond appropriately if a patient initiates.

<u>Response and revision</u>:
We understand that the labeling of the A20 code is confusing. While this code had been used for euthanasia which is by definition at the patient's request, the Dutch College of General Practitioners (DCGP / NHG) advised GPs to use this code to record any ACP conversation, and this was regardless of the topic or who initiated it.
We revised "While A20 was labeled as a conversation about euthanasia, it was also used to record ACP conversations on other topics" to "*While the A20 ACP code had originally been used for conversations on euthanasia, the Dutch College of General Practitioners advised to use it to record any ACP conversation.*[34-35]" (lines 129-131)

10. The conclusion refers to policy initiatives stimulating implementation of ACP in chronic progressive disease. It would strengthen the discussion if the authors could provide an example or two. Maybe there is some evidence in the literature of initiatives that have been successful in other countries? This could be taken into the discussion section with some implications/recommendations for practice and policy, as currently the recommendation is quite general.

<u>Response and revision</u>:
We agree with the reviewer that the recommendations can be improved by providing examples for clinical practice. We have therefore added some examples to the Conclusions section (revisions in italics):
Dutch GPs initiate ACP less frequently for persons with dementia, stroke and organ failure, compared to persons with cancer. Considering the complexity of initiating ACP in persons with organ failure or dementia, general practitioners may prioritize offering it to them and their family caregivers. ~~Policy~~ Practice improvement initiatives should stimulate implementation of ACP with chronic-progressive disease, *for example by reimbursing time to conduct ACP conversations. Also, guidelines addressing the treatment of the chronic progressive diseases can pay more attention to ACP. Already available tools to support healthcare professionals in addressing palliative care needs can be helpful as well*[59,60]*. [lines 357-361]*

All references included in this response can be found in the reference list of the manuscript.

VERSION 2 - REVIEW

**Reviewer**	**1**
**Name**	**Stevens, Julie**
**Affiliation**	**Vrije Universiteit Brussel**
**Date**	**10-Sep-2025**
**COI**	

Many thanks to the authors for their thorough and reasoned responses to my comments, which have adequately addressed my remarks. I did see that the study flow (page 31) is missing a label now but this is very minor; otherwise I have no additional remarks.
